# Translating the InChI: adapting neural machine translation to predict IUPAC names from a chemical identifier

**DOI:** 10.1186/s13321-021-00535-x

**Published:** 2021-10-07

**Authors:** Jennifer Handsel, Brian Matthews, Nicola J. Knight, Simon J. Coles

**Affiliations:** 1 Scientific Computing Department, Science and Technology Facilities Council, Didcot, OX11 0FA UK; 2 School of Chemistry, Faculty of Engineering and Physical Sciences, University of Southampton, Southampton, SO17 1BJ UK

**Keywords:** seq2seq, InChI, IUPAC, Transformer, Attention, GPU

## Abstract

We present a sequence-to-sequence machine learning model for predicting the IUPAC name of a chemical from its standard International Chemical Identifier (InChI). The model uses two stacks of transformers in an encoder-decoder architecture, a setup similar to the neural networks used in state-of-the-art machine translation. Unlike neural machine translation, which usually tokenizes input and output into words or sub-words, our model processes the InChI and predicts the IUPAC name character by character. The model was trained on a dataset of 10 million InChI/IUPAC name pairs freely downloaded from the National Library of Medicine’s online PubChem service. Training took seven days on a Tesla K80 GPU, and the model achieved a test set accuracy of 91%. The model performed particularly well on organics, with the exception of macrocycles, and was comparable to commercial IUPAC name generation software. The predictions were less accurate for inorganic and organometallic compounds. This can be explained by inherent limitations of standard InChI for representing inorganics, as well as low coverage in the training data.

**Supplementary Information:**

The online version contains supplementary material available at 10.1186/s13321-021-00535-x.

**Figure:**
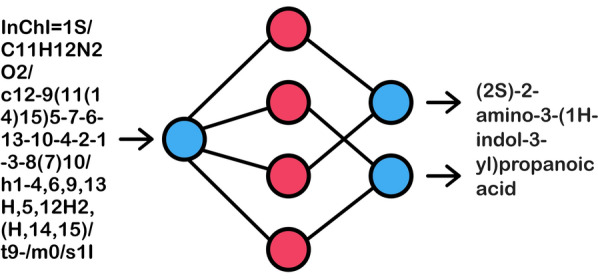

## Introduction

The International Union of Pure and Applied Chemistry (IUPAC) define nomenclature for both organic chemistry [1] and inorganic chemistry [2]. Their rules are comprehensive, but are difficult to apply to complicated molecules. Although there are numerous commercial software packages [3–6] that can generate IUPAC names from a chemical structure, these are all closed source and their methodology is unknown to the general public. Correctly generating IUPAC names is therefore an open problem, and in particular is an issue faced by synthetic chemists who want to give a standard name to a new compound. Although canonical SMILES [7] and InChI [8] can be used as identifiers, they are not designed to be human-readable, so the IUPAC name can be more informative identifier.

Neural networks excel at making general predictions from a large set of training data. They have shown great success in natural language processing, and have been deployed by Google on their online translation service [9]. Compared to earlier efforts that needed human-designed linguistic features, modern machine translation learns these features directly from matched sentence pairs in the source and target language. This is done with a sequence-to-sequence (seq2seq) neural network, made up of an encoder, which projects the input sentence into a latent state, and a decoder, which predicts the correct translation from the latent state.

A number of previous studies have applied sequence-based neural networks to cheminformatics. Schwaller [10] used a seq2seq recurrent neural network to predict the outcomes of chemical reactions, and other studies have presented generative models for automatic chemical design [11–14]. Two variants of SMILES have been proposed for use in machine learning [15, 16].

We present a seq2seq neural network that predicts the IUPAC name of a chemical from its unique InChI identifier. To our knowledge, there are two published machine learning models that predict IUPAC names from a SMILES string [17, 18], confirming our general methodology. However, we believe that our approach is more appropriate for deploying as a service, due to the ubiquitous use of InChI in online chemical databases. All standard InChI representations are generated with the official software from the InChI Trust [8], and although a normalized SMILES representation exists [19], it is not common in online databases. For practical applications, this means that a SMILES-based algorithm needs to be able to cope with the numerous equivalent SMILES representation for each molecule, but there is very little discussion of this point in the aforementioned studies.

## Methods

### Data collection

A dataset of 100 million SMILES-IUPAC pairs was obtained from PubChem [20], and the SMILES were converted to InChI with OpenBabel [21]. The average character length of the InChI identifiers was 134 ± 60, and 103 ± 43 for the IUPAC names. To simplify training, compounds were removed from the dataset if their InChI was longer than 200 characters, or their IUPAC name was longer than 150 characters. The resulting dataset of 94 million compounds was split into training data (90% of the data), with the remainder reserved for the validation and test sets. As IUPAC names of small molecules are usually easy to generate from procedural rules, validation and test sets were limited to compounds with an InChI length of 50 characters or greater. Due to the large volume of data available, the training set was reduced to a random sample of 10 million compounds. For the same reason, 10,000 samples were chosen for the validation set, and 200,000 were chosen for the test set.

### Experimental setup

**Fig. 1:**
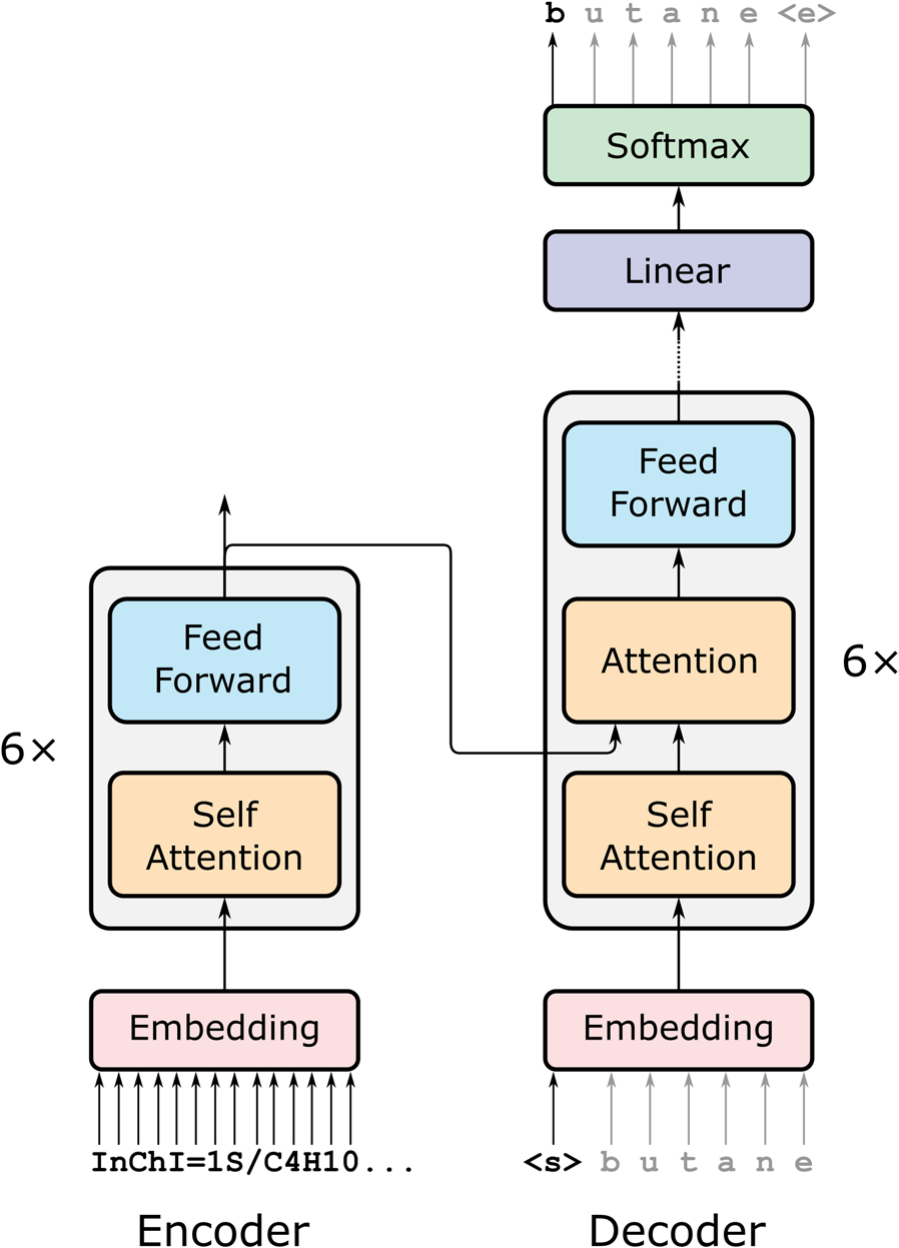
The encoder passes a numerical representation of the InChI to the decoder. The decoder is seeded with a start token, and its output is recursively re-input until it predicts an end token

All experiments were carried out with the PyTorch version of OpenNMT [22]. The final training script is included with the manuscript (Additional file 2), as are the InChI and IUPAC alphabets required for training (Additional files 3, 4). The neural network had a transformer encoder-decoder architecture [23], with six layers in both the encoder and decoder (Fig. 1). Each attention sub-layer had eight heads, and the feed-forward sub-layers had a hidden state size of 2048. Model weights were initialized with Glorot’s method [24].

The input (InChI) and target (IUPAC name) were tokenized into characters on-the-fly with OpenNMT’s pyonmttok module, with each character represented by a trainable embedding vector of length 512. Spaces were treated as separate tokens to enable detokenization of predicted names. The word vectors were augmented with positional encoding, to indicate the position of each character in the word. Character vocabulary was generated separately for InChI (66 characters) and IUPAC name (70 characters), using the whole training set. Both vocabularies included an out-of-vocabulary token.

The batch size was optimized for throughput: the optimal batch size was 4096 tokens which is equivalent to an average batch size of 30 compounds. Differing sample lengths within a batch were addressed by padding samples to a uniform length, and ignoring pad tokens when calculating model loss.

The model was regularized with a dropout rate of 0.1 applied to both dense layers and attentional layers [25]. This value was determined experimentally: increasing it above 0.1 reduced the test accuracy by ten percentage points, and training without dropout reduced the accuracy by one percentage point. The decoder output was regularized with label smoothing with magnitude 0.1 [26]. The model was optimized with the ADAM variant [27] of stochastic gradient descent, with beta_1 = 0.9 and beta_2 = 0.998. The learning rate was increased linearly to 0.0005 over 8000 warmup steps, then decayed with the reciprocal square root of the iteration number [23]. Gradients were accumulated over 4 batches before updating parameters.

The loss function to be minimized was the standard cross-entropy loss averaged over all tokens in the batch, defined as

1 $$\ell = \frac{1}{N}\sum\limits_{{{\text{batch}}}} {c \in } \sum\limits_{i} p \left( {c_{i} } \right)~{\text{log}}\left( {\frac{1}{{q\left( {c_{i} } \right)}}} \right)$$

where *N* is the number of tokens in the batch, $p({c}_{i})$ is the ground-truth probability that token *c* is the *i*th character in the alphabet (regularized with label smoothing as described above), and $q({c}_{i})$ is the corresponding probability predicted by the model. We report this as perplexity, defined as

2 $$\wp = e^{\ell }$$

which can be interpreted as the predicted token distribution being, on average, as unreliable as a uniform distribution with $\wp$ branches.

During training, the model was validated every 3200 batches on a validation set of 10,000 samples, as this size was found to be large enough to be representative. All models were trained until the validation accuracy stalled for three consecutive periods. Both training and validation used teacher forcing to improve convergence: rather than feeding predictions recursively into the decoder, each output character was predicted based on the ground truth from previous timesteps [28]. Training took seven days on a Tesla K80 GPU, with throughputs of 6000 tokens/second (InChI) and 3800 tokens/second (IUPAC name).

We performed limited training on a subset of 1 million samples to determine appropriate model parameters, and trialed an LSTM architecture [28] before settling on the transformer architecture described above. We also tested byte-pair encoding [29] and unigram language models [30] for tokenizing the InChI and IUPAC names into common clusters of characters, but the resulting accuracy was far lower than achieved using character-level tokenization. We also found that we could train an accurate model to translate SMILES to the IUPAC name, but such a model did not generalize to alternative (but equivalent) SMILES representations.

## Results

**Fig. 2:**
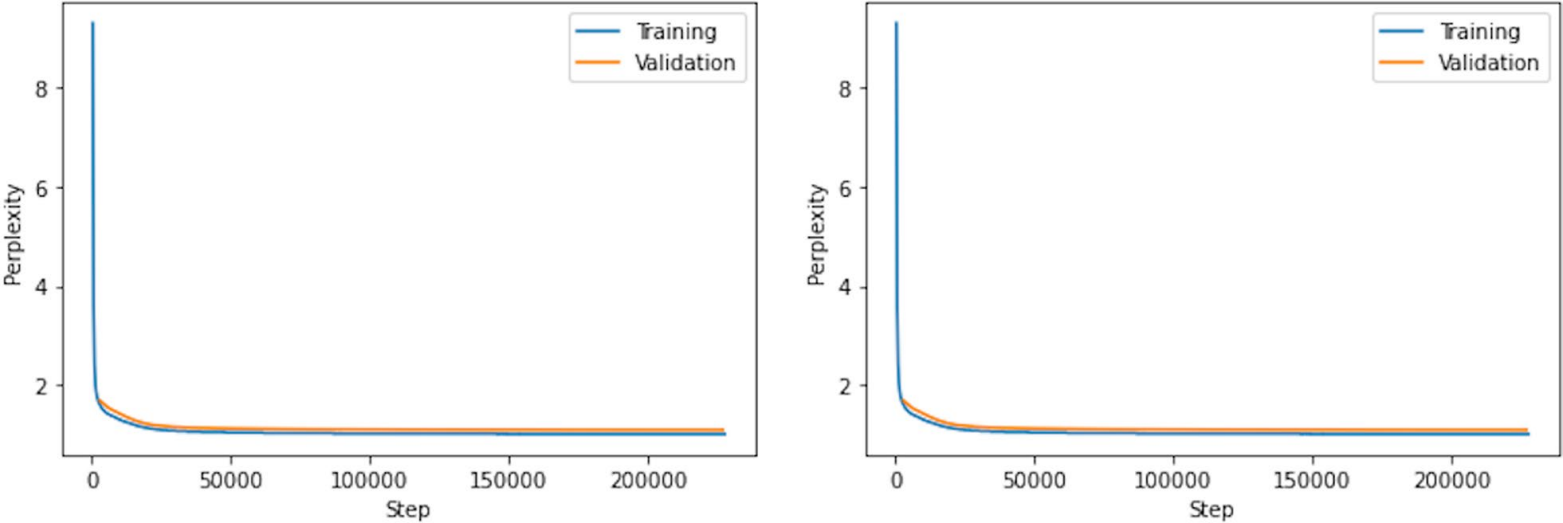
Perplexity and token accuracy during training of the InChI to IUPAC model

Training on 10 million samples converged with a validation perplexity of 1.09 (Fig. 2). We also found token accuracy to be a useful metric during training, defined as the proportion of correctly predicted characters over all IUPAC names in the training or validation set. At convergence, the validation token accuracy was 99.7%.

We evaluated the trained model on a test set of 200,000 samples. The most probable IUPAC name was found with a beam search (width 10) and a length regularizer of strength 1.0 [9]. To evaluate our test predictions, we report whole-name accuracy, which is the percentage of IUPAC names predicted without error. To quantify error in incorrect predictions, we use Damerau and Levenshtein’s [31–34] normalized edit distance, which measures distance (on a scale from 0 to 1) between two strings of characters, taking into account insertions, deletions and substitutions of a single character, as well as transposition of adjacent characters (Table 1). Although other authors [17] have used BLEU scores [35] for this purpose, we find this metric hard to interpret, as it has been parametrized specifically for natural language translation and scores vary greatly on reparametrization [36].

**Table 1:** Evaluation of the trained model on a test set of 200,000 molecules

Subset	Accuracy (whole name)	Normalized edit distance^a^
All	0.91	0.02 ± 0.03
Organic	0.91	0.02 ± 0.03
Inorganic	0.14	0.32 ± 0.20
Organometallic^b^	0.20	0.37 ± 0.24
Other organic–inorganic mixture	0.50	0.15 ± 0.18

^a^Average over subset, with dispersion indicated by mean absolute deviation

^b^Defined as having an explicit carbon–metal bond

**Fig. 3:**
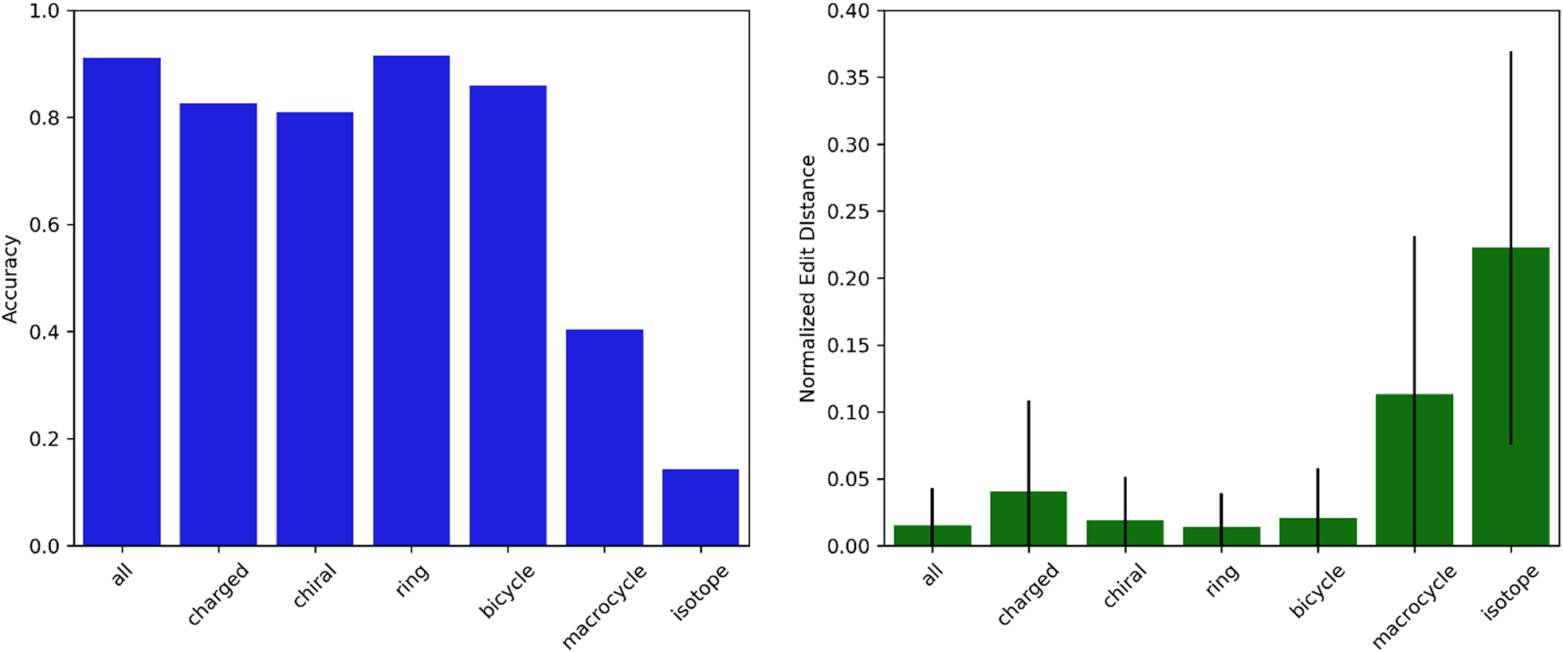
Accuracy of our model’s predictions on different subsets of organic molecules (left), and average normalized edit distances for the same subsets (right). Error bars indicate mean absolute deviation

Our model performs well on many classes of organic compound, with the exception of macrocycles and those with an isotopic substitution (Fig. 3). Edit distances suggest that, at least for organics, our predicted names are very similar to the ground truth even when incorrect. Our model is not appropriate for predicting the name of any compound with an inorganic constituent.

## Discussion

**Fig. 4:**
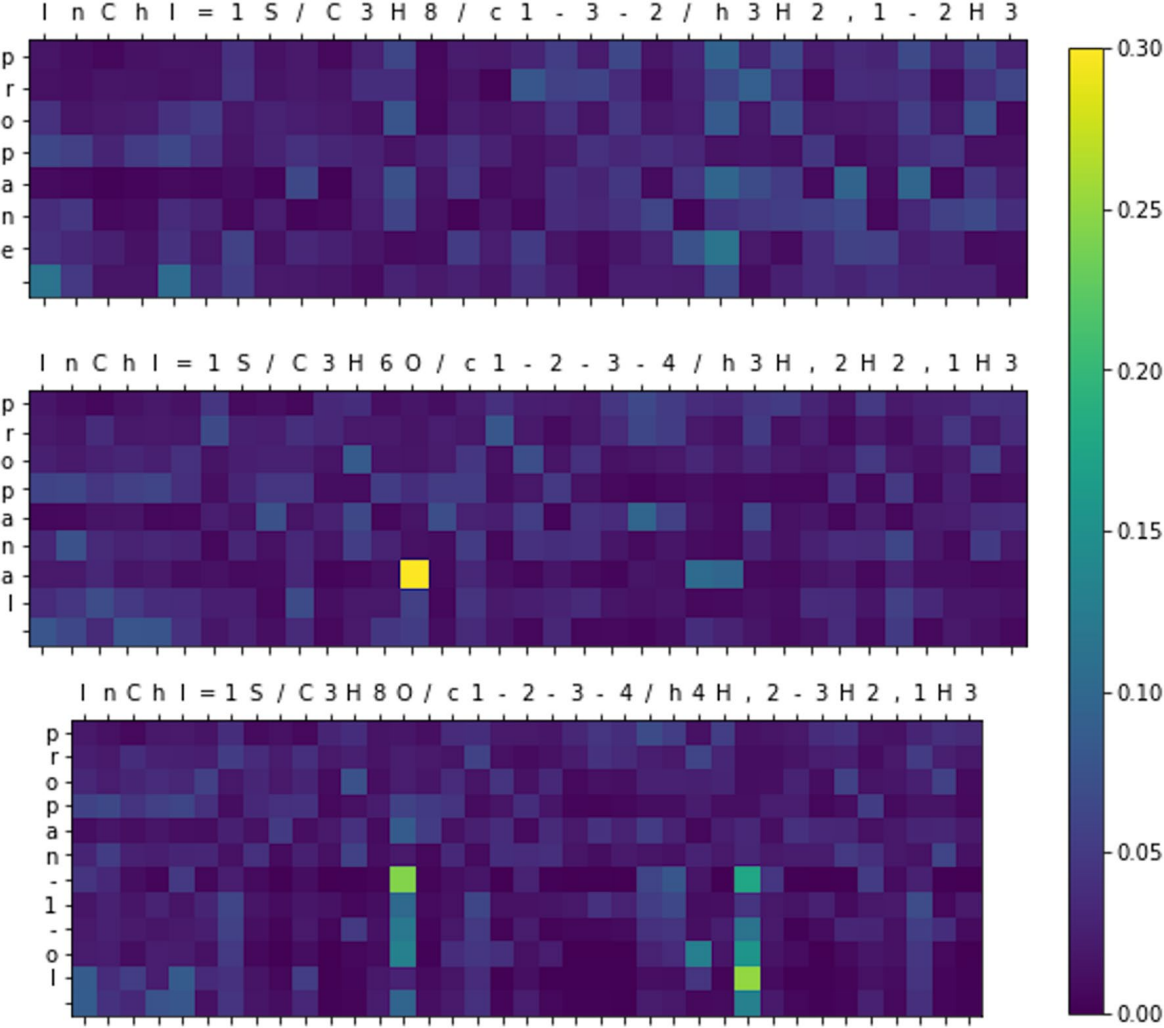
Attention coefficients from second to last layer of decoder, averaged over all heads, when predicting the names three similar molecules

The encoder-decoder architecture works by projecting the input InChI into a latent vector, and then predicting each character in the IUPAC name sequentially (conditioned on the previous predictions), until it predicts a stop token (Fig. 1). The attentional layer in the decoder essentially calculates a similarity between characters in the input to characters in the predicted IUPAC name. Visualizing these attention coefficients shows which parts of the input were important for predicting the output (Fig. 4).

All InChIs have three main layers with the chemical formula, connectivity, and hydrogen positions (in that order). When predicting the IUPAC name ‘propane’, no particular part of its InChI stands out in the attentional plot, but when predicting the suffix in ‘propanal’, the model pays attention to the oxygen element in the formula layer. Similarly, when predicting the ‘-1-ol’ suffix in propan-1-ol, the model pays particular attention to the oxygen atom (in the formula layer), and the fact that atom 4 (oxygen) has only one hydrogen (in the hydrogen layer).

We probed the model further by selectively setting characters in the InChI to the out-of-vocabulary token. As one might expect, mutating the ‘O’ in propan-1-ol changes the predicted IUPAC name to propane. But the model makes a correct prediction when all of the formula apart from the ‘O’ is mutated, presumably because the connectivity and hydrogen layers still make ‘propan-1-ol’ the most likely candidate (Table 2).

**Table 2:** Model predictions when mutating the InChI of propan-1-ol with an out-of-vocabulary token

InChI^a^	Predicted IUPAC name
InChI = 1S/C3H8O/c1-2–3-4/h4H,2-3H2,1H3	Propan-1-ol
InChI = 1S/C3H8**#**/c1-2–3-4/h4H,2-3H2,1H3	Propane
InChI = 1S/**####**O/c1-2–3-4/h4H,2-3H2,1H3	Propan-1-ol
InChI = 1S/C3H8O/c1-2–3-4/**####**2-3H2,1H3	Propan-1-one
InChI = 1S/C3H8O/c1-2–3-4/h4H,**#########**	Prop-1-en-1-ol
**#########**C3H8O/c1-2–3-4/h4H,2-3H2,1H3	Propan-1-ol

^a^Out-of-vocabulary token depicted with **#**

### Isomers

The InChI format specifies stereoisomerism with an optional layer. Our InChI to IUPAC model can successfully label enantiomers and diastereomers, even when their InChI differs by a single character (Table 3).

**Table 3:** Prediction of the IUPAC name of isomers not present in the training set

InChI	IUPAC name	Predicted IUPAC name
InChI = 1S/C15H13Cl3O4S/c16-10–1-4–13(5–2-10)23(20,21)9–12(19)8–22-15–6-3–11(17)7–14(15)18/h1-7,12,19H,8-9H2/t12-/**m1**/s1	(2R)-1-(4-chlorophenyl)sulfonyl-3-(2,4-dichlorophenoxy)propan-2-ol	(2R)-1-(4-chlorophenyl)sulfonyl-3-(2,4-dichlorophenoxy)propan-2-ol
InChI = 1S/C15H13Cl3O4S/c16-10–1-4–13(5–2-10)23(20,21)9–12(19)8–22-15–6-3–11(17)7–14(15)18/h1-7,12,19H,8-9H2/t12-/**m0**/s1	(2S)-1-(4-chlorophenyl)sulfonyl-3-(2,4-dichlorophenoxy)propan-2-ol	(2S)-1-(4-chlorophenyl)sulfonyl-3-(2,4-dichlorophenoxy)propan-2-ol
InChI = 1S/C20H18N2O/c1-23–20-15–9-8–10-17(20)16–21-22(18–11-4–2-5–12-18)19–13-6–3-7–14-19/h2-16H,1H3/**b21-16+ **	*N*-[(*E*)-(2-methoxyphenyl)methylideneamino]-*N*-phenylaniline	N-[(E)-(2-methoxyphenyl)methylideneamino]-N-phenylaniline
InChI = 1S/C20H18N2O/c1-23–20-15–9-8–10-17(20)16–21-22(18–11-4–2-5–12-18)19–13-6–3-7–14-19/h2-16H,1H3/**b21-16-**	*N*-[(*Z*)-(2-methoxyphenyl)methylideneamino]-*N*-phenylaniline	N-[(Z)-(2-methoxyphenyl)methylideneamino]-N-phenylaniline
InChI = 1S/C17H18N2/c1-12–3-5–16-14(7–12)9–18-11–19(16)10–15-8–13(2)4–6-17(15)18/h3-8H,9-11H2,1-2H3	(1S,9S)- / (1R,9R)-5,13-dimethyl-1,9-diazatetracyclo[7.7.1.0^2,7^.0^10,15^]heptadeca-2(7),3,5,10(15),11,13-hexaene^a^	5,13-dimethyl-1,9-diazatetracyclo[7.7.1.02,7.010,15]heptadeca-2(7),3,5,10(15),11,13-hexaene

^a^( ±)-Tröger's base

However, there are issues with predicting isomerism that are related to limitations in the InChI standard. InChI does not recognize optical activity in molecules with Nitrogen in a bridgehead position in a polycyclic system [37], such as Tröger's base, and as such the model cannot assign isomerism in these cases. Tröger's base also highlights the inability of our model to predict formatting such as superscripts.

### Charges, radicals and isotopes

The test set accuracy for charged molecules was 79%, and our model is able to predict the names of common charged organic species. However, due to low training set coverage, the model performs poorly when predicting the names of molecules with radicals or isotopic substitutions (Table 4). Although InChI encodes point isotopic substitutions with an extra layer at the end, our model tends to ignore this information and predict the name of the non-substituted compound. Similarly, our model predicts the names of molecules with a radical as if the radical were not present.

**Table 4:** Prediction of the IUPAC name of charged species, radicals, and molecules with isotopic substitutions

Common name	IUPAC name	Predicted IUPAC name
Phenolate	Phenolate	Phenolate
Ammonium	Azanium	Azanium
Trimethylammonium	Trimethylazanium	Trimethylazanium
Naphthalen-1-ylazanium	Naphthalen-1-ylazanium	Naphthalen-1-ylazanium
Methyl carbene radical	Methylene	Methane
Phenyl radical	Phenyl	Cyclohexatriene
Phenoxy radical	Phenyloxidanyl	Cyclohexa-2,4-dien-1-one
Heavy water	(^2^H_2_)Water	Deuteriooxydiazene
Tritiated water	(^3^H_2_)Water	Tritiooxytin
Deuterated benzene	(^2^H_6_)benzene	1,2,3,4,5,6-hexadeuteriobenzene
3-chloroalanine-Cl37	(^37^Cl)2-amino-2-chloroacetic acid	2-amino-2-chloroacetic acid

### Tautomers

Standard InChI can recognize certain tautomers [38], but when it does so, it encodes a general representation. This is powerful, but it does mean that information on the specific tautomer can be lost. We found that InChI does not standardize keto-enol tautomers or enamine-imine tautomers, and that our model can correctly predict the IUPAC name of specific tautomers in these cases (Table 5).

**Table 5:** Prediction of the IUPAC name of tautomers

Common names	IUPAC name	Predicted IUPAC name
Cyanamide (enamine-imine)	Cyanamide/methanediimine	Cyanamide / methanediimine
Glucic acid (keto-enol)	2-hydroxypropanedial / 2,3-dihydroxyprop-2-enal	2-hydroxypropanedial / 2,3-dihydroxyprop-2-enal
γ-Lactam (lactam-lactim)	pyrrolidin-2-one / 3,4-dihydro-2*H*-pyrrol-5-ol	pyrrolidin-2-one
Guanine	2-amino-1,7-dihydropurin-6-one	2-amino-1,7-dihydropurin-6-one
Guanine^a^	N/A	2-amino-1,7-dihydropurin-6-one
Oxazolium (mobile proton)	4,5-dihydro-1,3-oxazol-3-ium	4,5-dihydro-1,3-oxazol-3-ium

^**a**^Alternative resonance structure specified with fixed H layer

However, for simple proton shifts, InChI encodes the structure in the general form. For γ-lactam/γ-lactim tautomers, our model predicted the name of the lactam form. A similar effect can be seen with resonance forms of the five-membered ring in guanine. While it is possible to specify the resonance form with a non-standard fixed hydrogen layer [8], there were no such examples in our training set. Our model tends to ignore any fixed hydrogen layer: for example, it predicts the standard IUPAC name for guanine even when an alternative tautomer is specified. The same can be seen on charged species with a mobile proton: the oxazolium ion can have a protonated oxygen or nitrogen, but standard InChI does not specify the charge center and standardizes the location of the proton.

Overall, our model performed well on the limited range of tautomers we tested, considering the limitations of standard InChI.

### Inorganic and organometallic compounds

Our model performed poorly on inorganics, organometallics, and inorganic–organic mixtures (Table 1). This is partly because less than 2% of the training data fit in these categories, but also because standard InChI is inherently limited in representing complexes and organometallics. InChI was designed to provide a unique identifier, not a lossless structural representation, and the standard InChI ignores connectivity between carbon and metal bonds [38]. While it is possible to add a reconnected layer to represent organometallic bonds [8], this layer is not part of the standard specification, and is rarely used in online databases.

Furthermore, coordination complexes are difficult to represent accurately with commonly used chemical data formats. Neither InChI, SMILES nor the ubiquitous MOL v2000 structure format [39] is able to represent dative bonds, which means that even rules-based software packages struggle to predict correct IUPAC names. An extension to SMILES [40] and the MOL v3000 format [39] do allow such bonds to be specified, but are not widely used in public-access databases, making it difficult to build an appropriate training set.

We also found that many of the IUPAC names of inorganic and organometallic compounds in the PubChem database were inaccurate, with metal atoms disconnected (suggesting that the names had been generated from InChI). The result is that our model fails to predict the correct IUPAC name of many common inorganic and organometallic compounds (Table 6).

**Table 6:** Prediction of the IUPAC name of inorganic and organometallic compounds

Common name	IUPAC name^a^	Predicted IUPAC name
Ferrocene	Bis[(1,2,3,4,5-η)-cyclopentadienyl]iron	Cyclopenta-1,3-diene;iron(2 +)
Ferrocene^b^	Bis[(1,2,3,4,5-η)-cyclopentadienyl]iron	Cyclopenta-1,3-diene;1,2,3,4-tetrafluorocyclopenta[b]pyrrol-4-ide;iron(2 +)
Hexaamminecobalt(III) chloride	Hexaamminecobalt(III) chloride	Azane;trichlorocobalt
Cobalt tricarbonyl nitrosyl	Tricarbonylnitrosylcobalt	Carbon monoxide;nitroxyl anion;cobalt
Methylmagnesium bromide	Bromo(methyl)magnesium	Magnesium;carbanide;bromide
n-butyllithium	Butyllithium	Lithium;butane

^a^From ChemSpider [41] as PubChem names were not accurate

^b^With non-standard reconnected InChI layer

### Comparison with commercial software

To our knowledge, there are four major commercial software packages that can generate IUPAC names from a structure [3–6] by procedurally applying the IUPAC rules. However, as the rules can be applied in different ways, there are multiple valid IUPAC names for the same substance [42]. Although the latest IUPAC nomenclature for organic chemistry [1] describes a method for choosing a preferred IUPAC name, it is incomplete and difficult to apply algorithmically. As a result, the names generated by commercial packages are often in disagreement [43].

PubChem generates its IUPAC names with OpenEye’s Lexichem, so we additionally compared our model to Advanced Chemical Development’s ACD/I-Labs, ChemAxon’s Marvin, and Mestrelab’s Mnova. As some of these packages do not support batch conversion, we restricted our comparison to a test set of 100 molecules (Additional file 1).

**Fig. 5:**
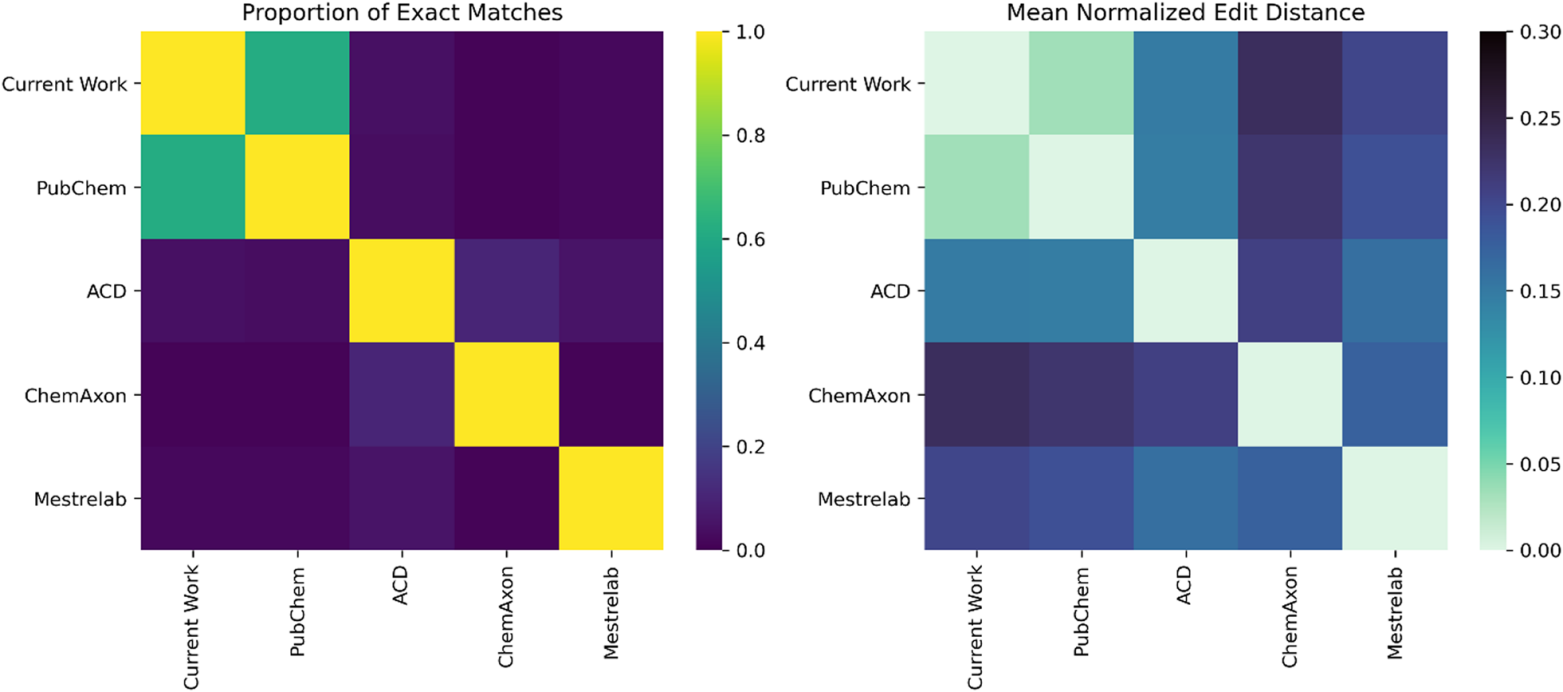
Comparison of IUPAC names from our model, PubChem, and commercial software packages, generated from a 100 molecule test set

We found that almost none of the names predicted by the commercial software were in agreement, and we calculated an average edit distance of 16–21% between the packages (Fig. 5). Many of the differences were due to differing conventions over the use of parentheses, but some names were substantially different (see supplementary information). Our predictions were close to the IUPAC names from PubChem, and although our model did not predict identical names to the three commercial packages, we found an average edit distance of 15% to 23%, similar to the variation between the software packages.

### Comparison with other machine learning models

Two similar machine learning models that predict IUPAC names from SMILES have emerged recently [17, 18]. We considered training our model on SMILES instead of InChI, but a disadvantage of using SMILES is the lack of a universal canonicalization scheme. Unless the model is trained to recognize equivalent SMILES strings, it must rely on a particular software package (often a particular version of that package) to standardize the SMILES. In comparison, InChIs are generated with official software from the InChI Trust [8], and there are no alternative implementations. The format was designed to provide a unique, consistent designation for each compound—in the most recent version of the software, the InChIs generated for the PubChem database had 99.99% agreement with those generated by the previous version [44].

Our model achieved a test set accuracy of 91%, while Rajan achieved an accuracy of 72% with a gated recurrent network trained on 60 million samples [17]. Krasnov reports an accuracy of close to 99% for their SMILES to IUPAC transformer network [18], however they only saw such high accuracy on SMILES strings of length 50 and lower. Our test set was limited to InChI strings of length 50 and greater to reflect the intended use of the model, and we do not have full access to Krasnov’s model to perform a fairer comparison.

Qualitatively, Krasnov’s model makes predictions that are similar to ours as it was also trained on data from PubChem. However, we are unable to make a detailed comparison beyond the examples in Table 7, as their full model has not been released. Rajan’s model is comparable to our model for medium-sized molecules, but has a tendency to get stuck in a repetitive cycle for more complicated molecules (Table 7). Furthermore, it is unable to predict stereochemistry as it relies on Chemistry Development Kit’s [45] canonical SMILES format, which removes stereochemistry markers. Interestingly, neither Krasnov’s nor Rajan’s model were able to predict the names of the most simple molecules, presumably because SMILES treats hydrogens implicitly, and the neural networks are unable to cope with such a short input.

**Table 7:** IUPAC name predictions for organic molecules of increasing complexity

IUPAC name	Predicted IUPAC name	Predicted IUPAC name (Rajan)	Predicted IUPAC name (Krasnov)^a^
Methane	Methane	Ethane	Methanidylmethane
Methanol	Methanol	2-methoxyethan-1-ol	Hydroperoxymethane
3-ethyl-1*H*-indole	3-ethyl-1H-indole	3-ethyl-1H-indole	3-ethyl-1H-indole
(2*S*)-2-amino-3-(1*H*-indol-3-yl)propanoic acid	(2S)-2-amino-3-(1H-indol-3-yl)propanoic acid	2-amino-3-(1H-indol-3-yl)propanoicacid	(2S)-2-amino-3-(1H-indol-3-yl)propanoic acid
(2*S*)-2-[[2-[[(2*S*)-2-amino-3-methylbutanoyl]amino]acetyl]amino]-3-hydroxypropanoic acid	(2S)-2-[[2-[[(2S)-2-amino-3-methylbutanoyl]amino]acetyl]amino]-3-hydroxypropanoic acid	2-[2-(2-amino-3-methylbutanamido)acetamido]-3-hydroxypropanoicacid	(2S)-2-[[2-[[(2S)-2-amino-3-methylbutanoyl]amino]acetyl]amino]-3-hydroxypropanoic acid
1,1-dicyclohexylethyl 5,6-ditert-butylbicyclo[2.2.1]heptane-2-carboxylate	1,1-dicyclohexylethyl 5,6-ditert-butylbicyclo[2.2.1]heptane-2-carboxylate	1-yl)-2-yl)-2-yl)-2-yl)-2-yl)-2-yl)-2-yl)-2-yl)-2-yl)-2-yl)-2-yl)-2-yl)-2-yl)-2-yl)-2-yl)-2-yl	1,1-dicyclohexylethyl 5,6-ditert-butylbicyclo[2.2.1]heptane-2-carboxylate
4-[2-[[(2*R*)-2-hydroxycyclopentyl]amino]-8-(2,3,6-trifluoroanilino)purin-9-yl]cyclohexane-1-carboxamide	4-[2-[[(2R)-2-hydroxycyclopentyl]amino]-8-(2,3,6-trifluoroanilino)purin-9-yl]cyclohexane-1-carboxamide	2–2-yl)-8-yl)-8-carboximidoyl-8-yl)-8-carboximidoyl-8-yl)-8-carboximidoyl-8-yl)…	4-[2-[[(2R)-2-hydroxycyclopentyl]amino]-8-(2,3,6-trifluoroanilino)purin-9-yl]cyclohexane-1-carboxamide

^a^Predicted through web interface [46]

As Rajan’s model is freely available for download, we were able to make predictions on a SMILES version of our 200,000 molecule test set, with inorganics removed (as their model was trained on organics only). We found that just 11% of Rajan’s predictions matched ours exactly, although this low number is to be expected as their model was trained on IUPAC names from ChemAxon, not PubChem. The average edit distance was 32%, which is nine percentage points higher than the corresponding distance between our model’s predictions and the names generated directly with ChemAxon that were discussed in the previous section.

## Conclusions

This work reinforces the recent interest in developing a machine learning based method for generating IUPAC names from a structure or a common chemical identifier. Our InChI to IUPAC model works very well for organics, but has some clear shortfalls, mainly due to known limitations of InChI and the composition of our training data. It is suitably robust to be deployed as a service, as long as predictions are constrained to organics. Our model will be integrated into ProperSea (https://psds.ac.uk/propersea), a physical properties platform that is be part of the United Kingdom’s Physical Sciences Data-science Service.

The strength of our model is that it translates InChI—a standardized identifier that is ubiquitous in open access chemical databases—to the IUPAC name. This simplifies the problem substantially, as there is no need to train the model to recognize different but equivalent representations of the same molecule, or rely on additional software to convert to a canonical representation. One downside is that there are limitations to standard InChI, meaning our model cannot predict accurate names for molecules with an inorganic component. It may be possible to address this issue by using a training set of non-standard InChIs with a reconnected metal layer.

Our comparison with commercial packages was inconclusive. The IUPAC rules for nomenclature are essentially an informally-specified algorithm that is inherently underdetermined, as the rules can be applied in different ways. It is therefore unsurprising that each commercial software package predicts different IUPAC names, but it is beyond the scope of this paper to explore this inconsistency in detail. Suffice to say that the discrepancy between our model and the commercial packages was similar to the discrepancy between the packages, and that these packages share many of the same limitations on generating names of inorganic compounds. IUPAC are still developing their nomenclature, and there is still work to be done to translate it into a procedural algorithm that can reliably generate the preferred name.

## Supplementary Information

**Additional file 1.** 100 molecule test set including InChI and IUPAC names from PubChem, ACD /I-Labs, ChemAxon, Mestrelab and the transformer presented in the current paper.

**Additional file 2.** Training script for OpenNMT-py.

**Additional file 3.** InChI character vocabulary needed for training the model with OpenNMT.

**Additional file 4.** IUPAC character vocabulary needed for training the model with OpenNMT.

## Data Availability

Our trained model is freely available for download under the Creative Commons license [47]. The dataset of 100 million compounds can be obtained from PubChem’s [20] public ftp server [48] in two separate files (CID-SMILES.gz and CID-IUPAC.gz). The two files were combined by merging on the CID column (the internal identifier used by PubChem) with GNU join [49]. The integrity of the SMILES data was verified with Open Babel 3.1.1 [21], which is freely available under the GNU General Public License. This was done by reading in each SMILES string and excluding 132,421 structures that could not be parsed. The integrity of the IUPAC column was verified by excluding names with unbalanced parentheses, using a simple regular expression with Python 3.8.5. The SMILES from PubChem were converted to InChI and canonical SMILES using Open Babel’s pybel module. The neural machine translation software, OpenNMT-py 2.0.0 [22], is freely available under the MIT license. Our training script and vocabularies for OpenNMT are included as supplementary information.

## Abbreviations

LSTM: Long short-term memory
seq2seq: Sequence to sequence
InChI: International Chemical Identifier
SMILES: Simplified molecular-input line-entry system
IUPAC: International Union of Pure and Applied Chemistry
